# Gut-derived imidazole propionate promotes atherosclerosis through myeloid imidazoline-1 receptor signaling: new biomarker and therapeutic target

**DOI:** 10.1038/s41392-025-02440-3

**Published:** 2025-10-27

**Authors:** Weiqi Wang, Dilvin Semo, Rinesh Godfrey

**Affiliations:** Vascular Signalling, Molecular Cardiology, Department of Cardiology I-Coronary and Peripheral Vascular Disease, Heart Failure, University Hospital Münster, Münster, Germany

**Keywords:** Cardiovascular diseases, Inflammation

In a groundbreaking study published in *Nature*, Mastrangelo et al. identified imidazole propionate (ImP), a gut microbiota-derived metabolite, as both a driver and therapeutic target in atherosclerosis.^1^ This discovery opens new avenues for early diagnosis and personalized cardiovascular treatment.

Cardiovascular disease remains the leading cause of mortality worldwide, with atherosclerosis serving as the primary pathological basis. Traditional risk factors such as cholesterol levels, hypertension, and smoking have guided prevention and treatment strategies for decades. However, many patients continue to experience cardiovascular events despite optimal management, prompting exploration of novel pathogenic mechanisms beyond conventional paradigms.

ImP is produced by intestinal bacteria through histidine metabolism; an essential amino acid obtained from diet. While linked previously to metabolic disorders including type 2 diabetes and insulin resistance,^2^ its role in cardiovascular disease was unclear. Notably, a recent study by Nageswaran et al. showed elevated ImP impairs endothelial cell function by reducing migration, angiogenesis, and vascular repair, promoting inflammation critical to atherosclerosis development.^3^ Their work highlights the contribution of ImP to endothelial dysfunction, complementing the emerging view of ImP as a multifaceted driver of atherosclerosis. Building on this, Mastrangelo et al. provide compelling evidence that ImP promotes atherosclerosis through activation of the imidazoline-1 receptor (I1R) on immune cells, identifying both biomarker and therapeutic target roles.

The authors combined mouse models and human investigations across two cohorts. Untargeted metabolomics in atherosclerosis-prone mice linked ImP strongly to disease. Remarkably, ImP administration to mice on a normal diet induced atherosclerotic lesions independently of cholesterol levels, challenging lipid-centric paradigms. These findings were validated in two human cohorts: the PESA (Progression of Early Subclinical Atherosclerosis) study with 400 participants and the IGT (Impaired Glucose Tolerance) cohort with over 1,800 participants, where plasma ImP levels were significantly higher in individuals with subclinical atherosclerosis. ImP strongly associated with metabolically active atherosclerosis, detected by 18F-fluorodeoxyglucose PET imaging, indicating early-stage disease. Importantly, ImP added diagnostic value beyond LDL cholesterol and high-sensitivity C-reactive protein (hs-CRP) (Fig. 1).

**Fig. 1:**
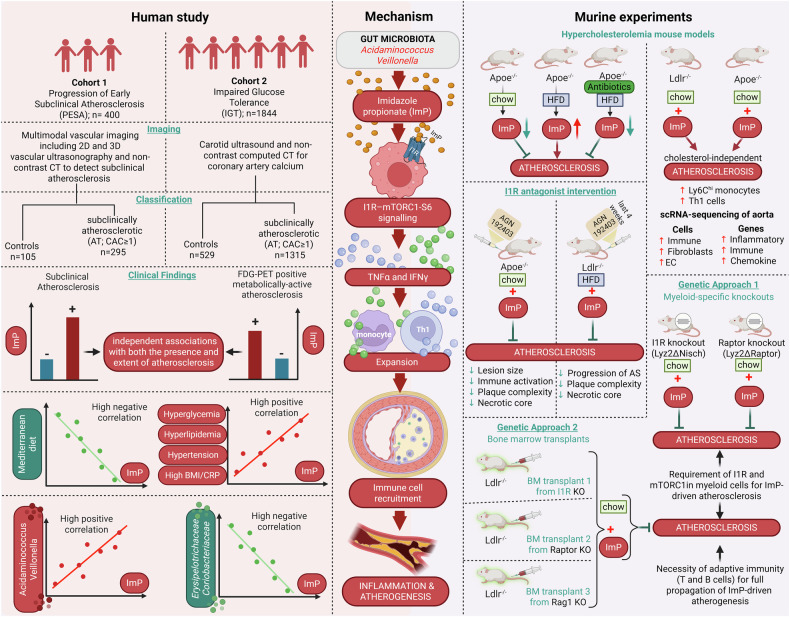
Insights from human and murine studies reveal gut microbial imidazole propionate as a causal driver of atherosclerosis. The figure illustrates experimental workflows establishing ImP as a causal driver and biomarker of atherosclerosis. Left panel: Human studies in PESA (*n* = 400) and IGT (*n* = 1844) cohorts used vascular imaging and plasma metabolomics to identify subclinical atherosclerosis. ImP levels were elevated in atherosclerosis, independent of traditional risk factors, providing additive diagnostic value. ImP correlated with adverse metabolic profiles and distinct gut microbiota signatures. Right panel: Mechanistic studies in atherosclerosis-prone mice demonstrated that ImP administration promoted lesions independent of cholesterol through inflammation via the ImP–I1R–mTORC1 axis in myeloid cells. Genetic deletion of I1R or mTORC1, or pharmacological inhibition with AGN192403, prevented atherogenesis. Center panel: Mechanistic pathway showing gut-derived ImP binding I1R on myeloid cells, activating mTORC1 signaling, driving pro-inflammatory cytokine production, and promoting leukocyte recruitment to arterial walls, resulting in cholesterol-independent plaque formation. The figure has been created with BioRender.com

Mechanistic studies revealed that ImP binds the imidazoline-1 receptor (I1R or nischarin) on myeloid cells, activating the mTOR (mechanistic target of rapamycin) signaling pathway. mTOR signaling plays complex roles in cardiovascular disease; mTORC1 activation promotes atherosclerosis while mTORC2 is protective. Selective deletion of I1R in myeloid cells (Lyz2ΔNisch mice) completely prevented ImP-induced atherosclerosis. Similarly, mice lacking the mTORC1 component Raptor in myeloid cells (Lyz2ΔRaptor mice) resisted proatherogenic effects of ImP, confirming the critical I1R–mTORC1 axis. Pharmacological blockade using a selective I1R antagonist (AGN192403) recapitulated these protective effects, reducing immune activation, plaque complexity, necrotic cores, and atherosclerosis progression in multiple mouse models without altering cholesterol. Moreover, treatment with AGN192403 reduced the increase of pro-inflammatory Ly6C^hi^ monocytes and T helper 1 (Th1) cells in the bloodstream, accompanied by a decrease in systemic cytokines such as TNF and interferon gamma. The receptor inhibition led to a significant reshaping of the immune environment that drives the development of atherosclerosis. These results firmly establish the ImP–I1R–mTORC1-S6 pathway in myeloid cells as a key atherosclerosis driver and a promising therapeutic target (Fig. 1).

The discovery of ImP as a biomarker and potential driver of atherosclerosis underscores the growing role of precision medicine in cardiovascular care. Current methods for early detection rely on costly, limited-access imaging, whereas a simple blood test for ImP could enable earlier identification of at-risk individuals and timely intervention. Critically, ImP represents the first immune-metabolism-mediated biomarker operating independently of traditional lipid pathways, offering distinct mechanistic approaches beyond LDL cholesterol and hs-CRP. Notably, ImP levels were lower in individuals following Mediterranean-style diets rich in fish, vegetables, and whole grains, while higher levels correlated with increased abundance of bacterial genera such as Veillonella and Acidaminococcus. However, high-protein Western diets rich in histidine may elevate ImP independent of cardiovascular risk, potentially confounding risk stratification. These associations suggest that dietary or microbiome-targeted strategies could modulate ImP production and reduce cardiovascular risk.

ImP adds to a growing list of gut microbiome-derived metabolites implicated in cardiovascular disease, such as trimethylamine N-oxide (TMAO), which promotes atherosclerosis through cholesterol and platelet pathways, and short-chain fatty acids, which generally exert protective effects.^4,5^ Unlike TMAO, ImP appears to act primarily through immune activation and inflammatory signaling, expanding our understanding of the gut–aorta axis in disease pathogenesis.

While functional evidence supports I1R activation by ImP, future studies should confirm direct binding through biochemical approaches such as co-immunoprecipitation or surface plasmon resonance and evaluate selectivity of AGN192403 across imidazoline receptor subtypes (I2R, I3R) to exclude off-target effects that could complicate therapeutic translation. Additionally, determining whether ImP acts as a direct I1R ligand or through intermediate molecular effectors will clarify the mechanistic pathway. Combining I1R antagonists with existing therapies such as statins may offer additive benefits, as ImP-mediated immune activation operates independently of lipid-lowering mechanisms. Compared to TMAO inhibition, ImP-targeting offers distinct advantages through direct immune modulation, potentially providing broader anti-inflammatory benefits.

The translation of these findings into clinical practice will require several key developments. First, standardised assays for ImP measurement must be developed and validated across diverse populations, particularly in Asian and African populations where distinct gut microbiota could alter predictive accuracy. Clinical cutoffs must be established based on PESA/IGT data analysis. Preliminary analysis of the PESA and IGT cohorts reveals inter-cohort variation in ImP concentrations (median ~29 nM in PESA vs. ~7 nM in IGT) and consistent association of upper-tertile ImP levels with increased atherosclerosis risk. Defining robust clinical cutoffs will require harmonized assay protocols and validation across diverse populations. Second, clinical trials will be needed to evaluate I1R antagonists as therapeutic agents. Comprehensive safety profiling is important, as chronic mTORC1 modulation may carry metabolic side effects. Third, interventional studies exploring dietary modifications or targeted microbiome manipulation could provide additional therapeutic options.

Despite these promising findings, several challenges remain. ImP biomarker utility in patients with gastrointestinal disorders presents complexity, as inflammatory bowel disease may artificially alter ImP levels, decoupling its atherosclerosis association. The relationship between ImP and cardiovascular outcomes needs validation in larger, more diverse populations. Standardized protocols should include dietary questionnaires and potentially fasting states to minimize dietary-induced fluctuations. Clinical trials are essential for validating I1R antagonists and assessing combination with existing therapies.

ImP as a cholesterol-independent driver and therapeutic target represents a significant advance in cardiovascular medicine. This reveals a novel immune-mediated pathway offering diagnostic opportunities alongside existing strategies. ImP may serve as a biomarker for detection and personalized intervention. Further research is needed to validate findings and develop therapies. Nageswaran et al. highlight the role of ImP in endothelial dysfunction,^3^ expanding its atherogenic impact. These studies underscore the potential of targeting gut microbial metabolites to prevent and treat cardiovascular disease, thereby advancing precision cardiometabolic care.

## References

[1] Mastrangelo, A. et al. Imidazole propionate is a driver and therapeutic target in atherosclerosis. *Nature*. **645**, 254–261 (2025). 10.1038/s41586-025-09263-w PMC12408353 40670786

[2] Koh, A. et al. Microbially Produced Imidazole Propionate Impairs Insulin Signaling through mTORC1. *Cell* **175**, 947–961 e917 (2018). 30401435 10.1016/j.cell.2018.09.055

[3] Nageswaran, V. et al. Gut Microbial Metabolite Imidazole Propionate Impairs Endothelial Cell Function and Promotes the Development of Atherosclerosis. *Arterioscler Thromb. Vasc. Biol.* **45**, 823–839 (2025). 40143816 10.1161/ATVBAHA.124.322346 PMC12017598

[4] Zhu, W. et al. Gut Microbial Metabolite TMAO Enhances Platelet Hyperreactivity and Thrombosis Risk. *Cell* **165**, 111–124 (2016). 26972052 10.1016/j.cell.2016.02.011 PMC4862743

[5] Wang, Z. et al. Gut flora metabolism of phosphatidylcholine promotes cardiovascular disease. *Nature* **472**, 57–63 (2011). 21475195 10.1038/nature09922 PMC3086762

